# Modulating Effects of Zingiberaceae Phenolic Compounds on Neurotrophic Factors and Their Potential as Neuroprotectants in Brain Disorders and Age-Associated Neurodegenerative Disorders: A Review

**DOI:** 10.3390/nu15112564

**Published:** 2023-05-30

**Authors:** Azraul Mumtazah Razak, Jen Kit Tan, Mazlina Mohd Said, Suzana Makpol

**Affiliations:** 1 Department of Biochemistry, Faculty of Medicine, Universiti Kebangsaan Malaysia, Kuala Lumpur 56000, Malaysia; 2 Faculty of Health Sciences, University College of MAIWP International, Kuala Lumpur 68100, Malaysia; 3 Centre of Drug and Herbal Research, Faculty of Pharmacy, Universiti Kebangsaan Malaysia, Kuala Lumpur 50300, Malaysia

**Keywords:** age-related ND, nerve growth factor, brain-derived neurotrophic factor, glial cell line-derived neurotrophic factor, Zingiberaceae, neurotrophins

## Abstract

The Zingiberaceae family possess various phenolic compounds that have significant systemic bioactivities in the brain, including in age-related neurodegenerative diseases. Neurotrophins are growth factors that protect neurons from oxidative stress, and dysregulation of the neurotrophic system may result in neurocognitive disease. Phenolic compounds from the Zingiberaceae family have been used in traditional and complementary medicine (TCM) to improve cognitive functions. These compounds may affect the expression of neurotrophic agents, but their underlying molecular mechanisms require further investigation. Therefore, the goal of this review is to determine the expression and functional roles of phenolic compounds from the Zingiberaceae family in brain disorders and age-related neurodegenerative disorders. While previous studies have proposed various mechanisms for the neuroprotective activity of these compounds, their precise mechanism of action remains complex and poorly understood. Despite some promising findings, there are still shortcomings in the therapeutic use of these herbs, and current interventions involving the Zingiberaceae family appear to be clinically insufficient. This article aims to summarize recent discoveries of phenolic compounds from several Zingiberaceae family members and their use as neuroprotectants and provide the first review of evidence-linked neuroprotective activity of bioactive ingredients from prominent members of the Zingiberaceae family.

## 1. Introduction

Phenolic acids are a major group of polyphenols. Plant-based diets that are high in bioactive compounds including polyphenols have demonstrated potential in reducing the risk of developing neurodegenerative disorders. In the past, researchers primarily studied the antioxidant effects of phenolic acids, which are due to their ability to chelate metals and scavenge free radicals [1,2]. However, recent studies have reported that bioactive polyphenols such as quercetin and demethoxycurcumin (DMC) can help prevent age-related cognitive decline associated with neurodegenerative diseases [3,4]. Polyphenols obtained from dietary sources can also improve cognitive deficits [5], promote synaptic plasticity [6], and enhance neurogenesis [7].

Zingiberaceae is a family of plants that includes ginger, turmeric, and galangal, among others. Some studies have suggested that phenolic compounds found in these plants may have potential therapeutic benefits for neurological brain disorders and neurodegenerative diseases [4]. For example, curcumin, a compound found in turmeric, has been studied for its potential neuroprotective effects in conditions such as Alzheimer’s disease [8], Parkinson’s disease [9], and multiple sclerosis [10]. Some studies have suggested that curcumin may help to reduce neuroinflammation and improve cognitive function [11]. Similarly, ginger has been studied for its potential to reduce inflammation and oxidative stress [12], which are believed to contribute to the development of neurological diseases. Studies have suggested that ginger may have neuroprotective effects in conditions such as Parkinson’s disease [13], Alzheimer’s disease [13], and Huntington’s disease [14]. It is important to note that while these studies are promising, more research is needed to fully understand the nature of brain and neurological disorders and the role of neuroprotectants, including those from Zingiberaceae family plants, in ameliorating the disease. Therefore, this review aims to summarize the mechanisms and main triggers of neurological and neurodegeneration diseases, and the neuroprotective properties of commonly reported phenolic compounds from Zingiberaceae family plants. This review also discusses the mechanisms and pathways by which the main phenolic compounds can exert their roles in the management of neurological brain disorders and neurodegenerative diseases.

### 1.1. Zingiberaceae Family and Their Phenolic Compounds

The Zingiberaceae family has culinary and medicinal uses and is widely distributed in the Indo-Malayan region [15]. One thousand six hundred species have been found in this family, which has about 53 genera including *Aframomum*, *Alpinia*, *Amomum*, *Boesenbergia*, *Curcuma*, *Elettaria*, *Etlingera*, *Hedychium*, *Hitchenia*, *Kaempferia*, *Renealmia*, and *Zingiber* [16,17]. The largest genus in the Zingiberaceae family is called *Alpinia* [18]. Their rhizomes are a source of phytochemicals which are beneficial for bioactivities [19].

**Figure 1:**
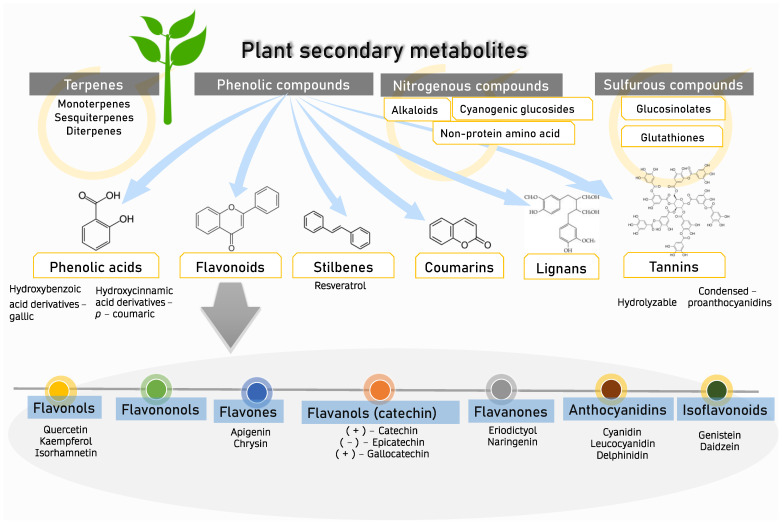
Plant secondary metabolites. Four groups of major secondary metabolites which can be found in plants. The focus of this review is on phenolic compounds (phenolic acids, flavanoids, stilbenes, coumarins, lignans, and tannins) and their flavonoid subset (flavonols, flavononols, flavones, flavanols, flavanones, anthocyanidins, and isoflavonoids).

A wide range of chemical substances are naturally produced by plants. Through numerous metabolic pathways, primary metabolites provide the necessary components for photosynthesis, translocation, and respiration. Secondary metabolites, created by biosynthetic modifications such as methylation, glycosylation, and hydroxylation, are typically the by-product of primary metabolites from various metabolic pathways and do not directly contribute to growth and development. Plant metabolites can be categorized into several main groups (Figure 1): (i) phenolic groups, (ii) terpenes, (iii) nitrogen-containing compounds, and (iv) sulfurous compounds [20]. The aromatic ring of phenolic compounds contains one or more hydroxyl groups. Phenolic acid, flavonoids, stilbenes, coumarins, lignans, and tannins are the principal naturally occurring phenolic compounds or phenolic antioxidants [21]. Approximately half of the naturally occurring plants are phenolics, whether present as a free state or as glycosides [22]. Flavonoids include flavonols, flavononols, flavones, flavanol (catechin), flavanones, anthocyanidins, and isoflavonoids [21]. These phenolic phytochemicals contribute to various biochemical properties, including the ability to regulate gene expression, act as antioxidant agents, and have antimutagenic and anticarcinogenic properties [23]. In addition, they reduce the risk of cardiovascular diseases [24,25,26] and are utilized in many aesthetic applications [27]. Among various Zingiberaceae family members, *Alpinia calcarata*, *A. malaccensis*, *A. nigra*, *Amomum aromaticum*, *A. maximum*, *A. koenigii*, *Curcuma amada*, *Curcumacaesia*, *C. picta*, *C. longa*, *Hedychiumcoronarium*, *Hedychium coccineum*, and *H. thyrsiforme* are the sources of many aesthetic compounds [19] and provide sustainable ecosystem applications [28]. *C. longa*, *C. xanthorrhiza*, and *Kaempferia pandurata* shield the skin against the effects of UV-B damage and possible skin wrinkles, and can act as antimicrobial, antiobesity, and anticancer agents [29,30,31]. Extraction of Zingiber rhizomes yielded phenolic acids, diarylheptanoids, terpenoids, sesquiterpene hydrocarbons, gingerols, shogaols, paradols, and terpenes. Asamenew et al. (2019) reported a comprehensive library of phytochemicals from ginger rhizomes [17]. Gingerols, shogaols, and paradols contribute to its recognized biological activities [32,33]. Some Zingiberaceae members have been discovered to contain essential oils such as pinene, limonene, eugenol, and geraniol [34]. Another team of researchers also identified trace levels of wikstromol, carinol, methyl-D-glucopyranoside, pyranone, propionate, and furanone [35]. Table 1 shows phenolic compounds of Zingiberaceae and their associated pharmacological activities. Phenolic compounds are categorized as primary antioxidants due to their redox characteristics. Phenolic antioxidants prevent oxidation by acting as free radical terminators or metal chelators. Phenolic antioxidants hinder oxidation; their primary role as free radical scavengers lessens oxidative stress, slowing down or preventing lipid oxidation or interrupting the propagation process, thus reducing the development of volatile decomposition products such as aldehydes and ketones [36]. Over time, oxidative stress may influence cognitive function and the establishment of pathogenic phenotypes and neurodegenerative disorders [37,38].

### 1.2. Zingiberaceae Family Plants Modulating Neurotrophic Pathways and Neuroinflammation

Neurological brain disorders and neurodegenerative diseases have complex and multifactorial causes and often involve a combination of genetic, environmental, and lifestyle factors. Imbalance of oxidative stress is also considered to be a potential trigger for neurological and neurodegenerative diseases [115]. Oxidative stress is harmful to healthy brain function and considered a causative factor for membrane hyperexcitability [116]. Accumulated reactive species can damage cellular macromolecules [117]. The production of O_2_^−^/H_2_O_2_ in neuronal brain redox signaling results in the formation of sulfenic acid [118]. Synaptic plasticity depends on Calcium (Ca^2+^) signaling. Therefore, the brain utilizes high amounts of adenosine triphosphate (ATP) to maintain its homeostasis. Due to the energy needs, the brain is susceptible to significant oxidative insults. The brain is high in lipid content and low in antioxidant capability [119]. There are two methods of antioxidant activity: prevention of reactive oxygen species (ROS) production and clearance of the damaged by-products of oxidative stress [120]. The first line of defense consists of enzymatic antioxidant disease, and the non-enzymatic antioxidant system serves as the second line of defense [121]. The repair systems engage by restoring oxidatively damaged macromolecules by stimulating the activities of phospholipases, peroxidases, or acryl transferases or eliminating them [122,123].

Nuclear factor erythroid 2-related factor 2 (NRF2) regulates antioxidant response. The actin-anchored protein Kelch-like ECH-associated protein 1 (Keap1), which is mostly located in the cytoplasm, and the transcription factor nuclear factor erythroid 2–related factor 2 (Nrf2) interact when cells are dormant [124]. When exposed to oxidative stress or substances that affect the cysteine residues in Keap1, Nrf2 is freed from ongoing degradation by dissociating from Keap1 and translocating into the nucleus to promote the activity of antioxidant enzymes and reduction of intracellular ROS [125]. Previously, in experimental models of amyotrophic lateral sclerosis (ALS) and multiple sclerosis (MS), acetyl-11-keto-beta boswellic acid (AKBA) stimulated the Nrf2/HO-1 pathway and promoted neuroprotection [126,127]. Neurodegenerative diseases lead to irreversible neuronal degeneration [128] which can be manifested by an increase in protein aggregates that encourage glial activation and inflammation, mitochondrial dysfunction, imbalance of neurotransmitters, and autophagia [129,130] further associated with neuroinflammation [116].

Numerous inflammatory processes are linked to neurocognitive diseases affecting the central nervous system (CNS) [131,132,133]. Additionally, extracellular damage-associated molecular pattern molecules (DAMPs) and inflammatogenic molecules are produced from damaged brain cells by cerebral inflammation, exacerbating CNS pathologies [134]. By eradicating or suppressing various infections, neuroinflammation serves as a protective defense system for the brain [135]. By encouraging tissue healing and clearing away cellular waste, this inflammatory response may be beneficial yet detrimental as a persistent inflammatory response might prevent regeneration [136]. Due to exogenous factors such as infection or drugs, or endogenous factors such as genetic mutation and protein aggregation, inflammation can be stimulated persistently [137]. Chronic activation of microglia leads to sustained production of proinflammatory cytokines that contributes to progression of various neurodegenerative diseases [138]. Under neurotoxic and neurodegenerative circumstances, reactive astrocytes can be polarized into different phenotypes which are determined by microglia [139]. Tumor necrosis factor-alpha (TNF-α) and interleukin-1 (IL-1) are examples of pro-inflammatory cytokines generated by activated microglia that induce A1 polarisation, while interleukin-10 (IL-10) promotes A2 polarisation [140]. Additionally, research has linked pathways associated with pain and inflammation [141,142]. Microglia and astrocytes are involved in persistent proinflammatory responses, which contribute to the development of neurodegenerative disorders [131,142]. Astrocyte polarisation has been reported in a number of neurodegenerative illnesses and neurotoxic circumstances, including ischemia [143], traumatic brain injury (TBI) [144], Alzheimer’s disease (AD) [145], and Parkinson’s disease (PD) [146]. Amyloid-β1-42 peptide (Aβ1-42) can activate microglial cells, which then in Alzheimer’s disease secrete pro-inflammatory chemokines and cytokines [147]. In addition, viral infections can stimulate astrocytes and microglia and subsequently trigger peripheral immune cells to invade the CNS, thus creating an inflammatory environment [148]. In mice with kainate-induced status epilepticus, microglia and astrocytes were sequentially activated [149]. Brain cells emit a variety of chemicals to deal with metabolic stressors. These chemicals including neurotrophic factors supporting neuronal survival and modulating the homeostasis of neuroinflammation [150].

Neurotrophic factors are proteins secreted by different cell types. They support neuronal cell development, survival, and neurite outgrowth. They perform crucial functions in the differentiation and proliferation of neurites and apoptosis [151]. There are three families of neurotrophic factors: The first is the NGF family, also known as neurotrophins. Several examples include nerve growth factor (NGF), brain-derived neurotrophic factor (BDNF), neurotrophin-3 (NT-3), and neurotrophin-4 (NT-4) also known as neurotrophins 5 (NT-5) (NT-4/5) [152]. Second are the glial cell line-derived neurotrophic factor (GDNF) family ligands including GDNF and neurturin (NRTN). The third group is a heterogeneous group of molecules that belong to the cytokine family [153]. In vivo study hypothesized that dysregulation of neurotrophic factors may be involved in the etiology of autism [154], anxiety [155], major depressive disorder (MDD) [156], post-traumatic stress disorder (PTSD) [157], schizophrenia [158], and various neurodegenerative disorders such as PD [159], AD [160], and bipolar disorder (BD) [161]. The stimulation of motor neurons with BDNF may improve motor function in an amyotrophic lateral sclerosis (ALS) model [162]. Another study verified the association of neurotrophic factors with neuroinflammation. They established that combined injury to mouse hippocampal neurons can release BDNF and speed up neuronal apoptosis [163]. In a proteomic profile, neurotrophic factors (BDNF and epidermal growth factor, EGF) were also reportedly implicated in neuroinflammation and blood-brain barrier (BBB) permeability in familial and sporadic ALS [164]. Depression and AD have been connected to polymorphisms in genes that change the level of neurotrophins [165]. In diabetic polyneuropathy, neuropathic pain and obesity are modulated by TrkB signaling. Thus, patients with diabetic polyneuropathy have significantly greater serum levels of TrkB and BDNF than healthy individuals [166]. NGF and PGE2 serum levels in the brain were positively associated with headache frequency in adult migraine patients, whereas BDNF and VEGF serum levels were not [167]. Moreover, a four-week period of persistent overexpression of BDNF, TrkB, and GDNF was associated with anticonvulsant effects [168]. The extent of the injury and genetic variation influence the expression of neurotrophins after traumatic brain injury (TBI) [152]. Furthermore, reduction in NGF-expressing neurons increases the risk of developing autism spectrum disorder (ASD) [169].

**Figure 2:**
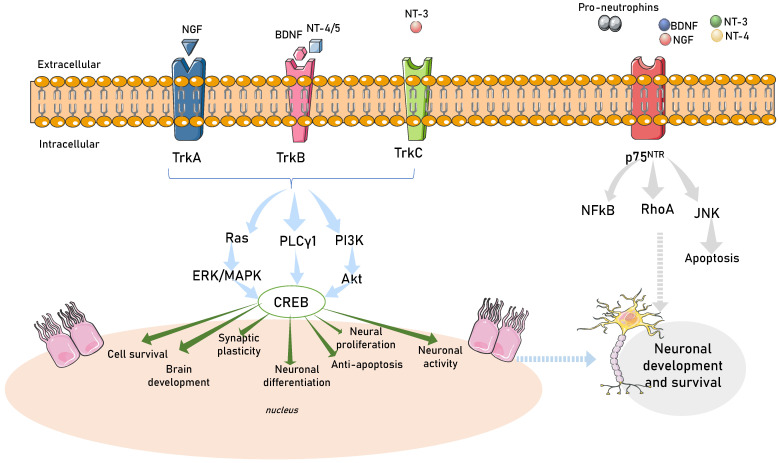
Neurotrophin intracellular signaling pathways. A schematic view of TRK receptor tyrosine kinases and major signal transduction pathways involved in cell survival, development, synaptic plasticity, neuronal differentiation, neural proliferation, and neuronal activity. Activity-dependent mechanisms act through activation of transcription mediated by CREB (calcium responsive element binding protein) in the nucleus of the cell. TRKA is activated by nerve growth factor (NGF), TRKB is activated by brain-derived neurotrophic factor (BDNF), neurotrophin 4/5 (NT-4/5) and TRKC are activated by neurotrophin-3 (NT3). RAS, rat sarcoma oncogene; ERK, extracellular-signal-regulated kinase; PI3K, phosphatidylinositol-4,5-bisphosphate 3-kinase; Ak strain transforming (Akt); tropomyosin-related kinase A (TrkA); tropomyosin-related kinase B (TrkB); tropomyosin-related kinase C (TrkC); phospholipase C-γ1 (PLCγ1); extracellular signal-regulated kinase/mitogen-activated protein kinase (ERK/MAPK); p75 neurotrophin receptor (P75NTR); nuclear factor-κB (NFKB); ras homolog family member A (RhoA); Jun N-terminal kinases (JNK).

Neurotrophins are produced in the nervous system, gastrointestinal tract, and urinary tract [170,171]. They mediate the differentiation and survival of neurons by activating downstream signaling pathways [172,173]. Neurotrophins are initially produced in a pro-form, then go through proteolytic cleavage to create mature neurotrophins. Neurotrophins possess stronger affinity for binding with Trk receptors compared with p75 neurotrophin receptors (p75NTR) [174]. With the capacity to transmit signals, the phosphorylated tyrosine residues then induce the recruitment of intracellular proteins. NGF is regulated by tropomyosin receptor kinase receptor A (TrkA), BDNF and NT-4/5 are primarily regulated by tropomyosin receptor kinase receptor B (TrkB), and NT-3 is primarily regulated by tropomyosin receptor kinase receptor C (TrkC) [175]. There is some overlap in cross-activations due to significant structural homology shared by Trk receptors and neurotrophins. Additionally, TrkB and TrkC have truncated isoforms. However, these truncated variants cannot evoke the same reaction since they lack cytoplasmic tyrosine kinase catalytic domains [176]. Upon binding with neurotrophins, Trk receptors then activate several intracellular signaling pathways (Figure 2).

#### 1.2.1. The Ras/ERK (MAPK) Pathway

The small, membrane-associated Ras proteins’ activity controls the Ras/ERK pathway. Shc binds to the phosphorylated tyrosine 490 and induces the signal activation cascade. Ras is then activated through guanosine triphosphate (GTP) binding [177]. Raf further activates MEK to phosphorylate ERKs [178]. ERK activation regulates neuronal enzymes and ion channels [179], neuritic process lengthening, and neuronal survival [180]. Depression can affect the activation of ERKs [181] and chronic pain is caused by hypersensitization of NGF [182]. Moreover, activation of JNK and p38 MAPK induced cerebral angiopathy [183].

#### 1.2.2. Phospholipase C, Gamma 1 (PLCγ1) Pathways

PLCγ1 mediates NT/Trk receptor interactions [184]. Tyrosine phosphorylation controls the PLC-beta protein. The SH-2 and SH-3 domains are two of the domains found in PLC. Once activated, lipid phosphatidylinositol 4,5-bisphosphate is cleaved by PLC-1 to produce inositol trisphosphate (IP3) and diacylglycerol (DAG) [185]. Since IP3 is soluble, it can diffuse and cause Ca^2+^ to be released into the cytoplasm [186]. Protein kinase C may be activated to compensate for the increase in Ca^2+^ or DAG concentration in the cells [187].

#### 1.2.3. The PI3K/Akt-mTOR Pathway

Trk receptor phosphorylation triggers PI3K activation. TrkA phosphorylates Shc, which then forms a complex with Grb2 to phosphorylate the Gab1 adaptor protein [188]. It also induces insulin receptor substrate 1 (IRS1) to become phosphorylated [189]. Mechanistic target rapamycin complex 1 or 2 (mTORC1 or mTORC2) is triggered by PI3K/Akt activation [190]. Promoting protein translation and synthesis, ribosomal biogenesis, and autophagy, the mTORC1 protein kinase complex is important for metabolism. It also controls mRNA translation by phosphorylating downstream effectors including P70 ribosomal S6 protein kinase (p70S6K) [191]. In addition, protein kinase B (Akt), which is linked to a number of clinical diseases, is one of the AGC kinases that are phosphorylated by mTORC2 to promote cell proliferation and survival and promote cytoskeleton remodeling [192].

#### 1.2.4. The p75NTR-Mediated Signaling Pathway

All neurotrophins have low affinity for the p75 neurotrophin receptor (p75NTR). Neurotrophins and each of their proforms are bound by the extracellular domain. The outcomes of p75NTR activation are influenced by interactions with other receptors. Due to the absence of enzymatic activity in its cytoplasmic domain, p75NTR does not signal via conventional channels. Therefore, interactors of the intracellular domain or interacting proteins that are attracted to or connected with the receptor contribute to the signaling of the p75NTR including apoptosis [193,194], p53 activation via the Jun kinase signaling cascade, and activation of NF-κB for neuronal survival [195] and axonal development [196]. p75NTR activation is also influenced by interactions with other receptors and transmembrane proteins [197,198]. p75NTR has a beneficial effect on cell survival by promoting ceramide production [199]. Ceramide controls ERK/MAPK, PI3K/Akt, Jun kinase, and NFkB signaling pathways [200].

Ginger’s 6-shogaol has been shown to have neuroprotective effects against H_2_O_2_-induced neuronal death in astrocytes by inhibiting ROS, Bax, and caspase 3 while increasing BDNF, GDNF, NGF, Bcl-2, and Bcl-xL via ERK1/2-mediated signaling [201]. In a mouse model of systemic neuroinflammation, a botanical mixture consisting of *Zingiber officinale* (150 mg kg^−1^), *Echinacea purpurea* (20 mg kg^−1^), and *Centella asiatica* (200 mg kg^−1^) demonstrated significant potential as an anti-inflammatory agent. Specifically, the study found that the density of the complement component 3 (C3) in the prefrontal cortex of lipopolysaccharides (LPS)-treated mice was significantly reduced following administration of the botanical mixture. This reduction in C3 density is indicative of anti-inflammatory activity and may contribute to the observed improvement in cognitive function seen in in vivo studies. Notably, the botanical mixture was found to be effective despite the persistently high level of C1q, which can modulate gene expression critical for neuronal survival when over-expressed in the absence of other complement components [202].

**Figure 3:**
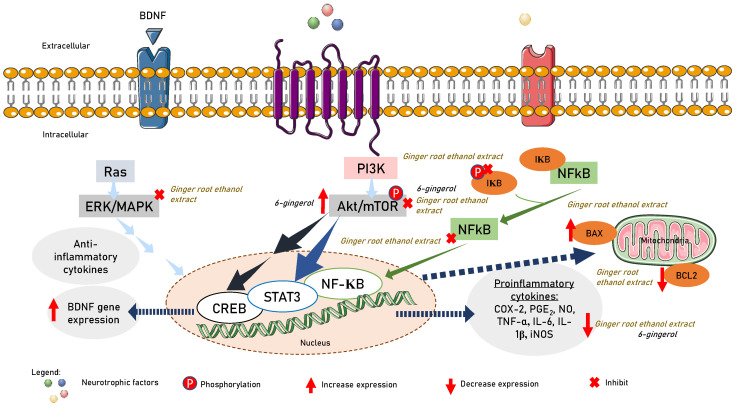
6-gingerol suppression and activation of Akt/STAT3 to suppress proinflammatory cytokine release and Akt/CREB to promote BDNF gene expression. Ginger root extract ameliorated microglia-mediated neuronal insults via upregulating the expression of Bax and reducing the expression of Bcl-2. Ginger root extract suppressed NF-κB and AKT/STAT3, and the MAPK pathway in the neuroinflammatory response.

Several pathways associated with neuroinflammation and neurodegeneration can be activated by matrix metalloproteinases (MMP)-2 and MMP-9. Natural compounds have been found to regulate signal transduction pathways, resulting in the downregulation of both MMP-2 and MMP-9 gene and protein expression. Although these effects may be attributed to their general anti-inflammatory and antioxidant properties, certain compounds have been directly shown to have anti-proteolytic effects on MMP-2 and/or MMP-9. Examples of such compounds include Ala–Thr–Pro–Gly–Asp–Glu–Gly (ATPGDEG), Leu-Ser-Gly-Tyr-Gly-Pro (LSGYGP), a naturally occurring *N*-farnesylated dibenzodiazepinone-BU-4664L, ageladine A, quercetin, and myricetin [203]. Phenolic compounds such as 6-gingerol, 8-gingerol, 10-gingerol, 6-shogaol, 6-paradol, zerumbone, curcumin, α-terpinyl acetate, curcumenol, and methoxyflavone have demonstrated beneficial effects on neurogenesis and neuroprotection, improved clinical symptoms in animal models, and reduced inflammatory mediators such as IL-1β, TNF-α, and IL-6 release [204,205,206,207,208]. Pre-treatment of SIM-A9 microglial cells (CRL-3265) with ginger root ethanol extract at a dose of 200 µg/mL showed neuroprotective effects by inhibiting the expression of cyclooxygenase (COX)-2 and inducible nitric oxide synthase (iNOS), which reduced the release of prostaglandin E2 (PGE2) and nitric oxide (NO). Additionally, ginger root ethanol extract has been shown to decrease the production of TNF-α and IL-6 induced by LPS. Ginger root ethanol extract also improved microglia-mediated neuronal damage by decreasing the expression of Bcl-2 and increasing Bax. As a result, ginger root ethanol extract was able to reduce neuroinflammation in LPS-stimulated mouse microglia by modulating serine-threonine protein kinase (Akt)-signal transducer and activator of transcription 3 (STAT3), Akt/STAT3, mitogen-activated protein kinases (MAPK), and NF-κB signaling pathways in the neuroinflammatory response [209]. Treatment with 6-gingerol at a dose of 5 mg/kg in male C57/BL6J mice and doses of 10, 20 and 30 μM of primary microglia from 1-day-old C57/BL6J mice showed improvements in cerebral ischemia injury by suppressing microglia-mediated neuroinflammation by downregulating the Akt-mammalian target of the rapamycin (mTOR)-STAT3 pathway [204]. On the other hand, oral administration of 10 or 25 mg/kg/day of 6-gingerol was reported to upregulate the protein levels of neurotrophic factor BDNF, apparently mediated by activation of the Akt-cAMP response element-binding protein (CREB) pathway [210]. Their crosstalk is summarized in Figure 3. Zerumbone and curcumin were found to reduce inflammation and provide neuroprotection by inhibiting the translocation of NF-κB into the nucleus, thereby reducing the activation of reactive oxygen species. Moreover, activation of the pathways of phosphatidylinositol-3/protein kinase B/glycogen synthase kinase-3 and phosphatidylinositol-3/protein kinase B/cAMP response element-binding protein/brain-derived neurotrophic factor was found to reduce neurodegeneration [211].

**Figure 4:**
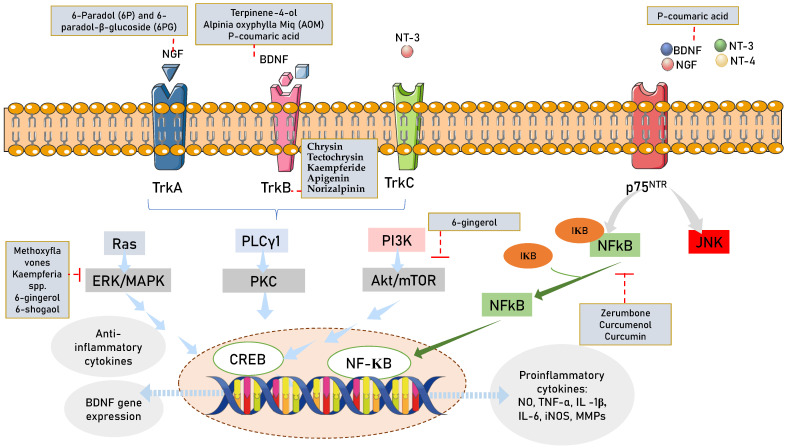
Zingiberaceae targeting neurotrophin intracellular signaling pathways. Legends for figure: Akt—Ak strain transforming; NGF—nerve growth factor; BDNF—brain derived neurotrophic nerve factor; TrkA—tropomyosin-related kinase A; TrkB—tropomyosin-related kinase B; TrkC—tropomyosin-related kinase C; NT3-neurotrophin 3; NT4/5—neurotrophin 4/5; PLCγ1—phospholipase C-γ1; ERK/MAPK—extracellular signal-regulated kinase/mitogen-activated protein kinase; CREB-cAMP—response element binding protein; P75NTR—p75 neurotrophin receptor; NFKB—nuclear factor-κB; RhoA—ras homolog family member A; JNK—c-Jun N-terminal kinases.

The basic fibroblast growth factor (bFGF)/NGF/TrkA/heat shock protein 70 (Hsp70) signaling pathway was reportedly modulated by *Afromomum* extracts [212], while the BDNF/TrkB/Akt signaling pathway was activated by Alpinia extract [213]. The CREB pathway [214] and the peroxisome proliferator-activated receptor-gamma coactivator (PGC)-1alpha (PGC1)/fibronectin type III domain-containing protein 5 (FNDC5)/BDNF pathway can both be activated by curcumin [215]. Additionally, *Kaempferia* spp. blocks autocrine IL-6/STAT3 signaling and decreases EGF-induced IL-6 synthesis [216]. A summary of beneficial effects of Zingiberaceae family members on neurogenesis and neuroprotection is presented in Table 2 and Figure 4.

### 1.3. Brain Disorders and Brain Insults

Brain disorders are medical conditions that can interfere with the normal functioning of the brain and can result in a variety of symptoms that impair cognitive, emotional, and physical abilities, depending on which areas of the brain or nervous system are affected. Some examples of brain disorders include neurodegenerative diseases such as Alzheimer’s and Parkinson’s diseases, mood disorders such as depression and bipolar disorder, anxiety disorders such as generalized anxiety disorder and panic disorder, schizophrenia, substance use disorders, developmental disorders such as autism spectrum disorder and attention deficit hyperactivity disorder (ADHD), stroke (caused by a blockage or bleeding in the brain), and traumatic brain injury. Stroke, ischemia, and traumatic brain injury are brain insults that are considered risk factors for developing neurodegenerative disorders. The degeneration of higher-order structural brain networks is commonly observed in cases of sub-acute ischemic stroke, and this breakdown of structural brain networks might play a role in the development of domain-specific cognitive impairments over time [237] contributing to age-related neurodegenerative disease.

### 1.4. Role of Zingiberaceae Family Plants in Managing Age-Related Neurodegenerative Disease

Specific brain regions were impacted more frequently in cases of cognitive impairment. Age-related alterations in neuroanatomical structures have been connected to cognitive function. A previous study indicated that geriatric cognitive performance is determined as early as the age of 20 [238]. Even though some cognitive abilities are relatively stable with ageing, those that depend on mental functioning deteriorate as people grow older [239]. The dentate gyrus (DG) of the hippocampal formation has the capacity to produce new neurons throughout the human lifetime. While pathological ageing, which is characterized by memory deficits, is linked to neurogenesis exhaustion, successful ageing, when it can preserve memory functions, is associated with the maintenance of a relatively high neurogenesis level [240,241,242]. The phenotypic age of the human brain, as revealed via deep learning of anatomic magnetic resonance images (MRI) reflects patterns of structural change related to cognitive decline, and the prefrontal cortex is the region of the greatest age-related vulnerability [243]. Whole ethanol extract of fresh *Zingiber cassumunar* rhizomes reduced neuronal cell loss in the hippocampus while suppressing the inflammatory response by reducing the expression of glial fibrillary acidic protein, GFAP (a marker of astrocyte activation) and IL-1ß in the hippocampus [244]. Another group of researchers reported that *Zingiber purpureum* Roscoe extract promotes neuronal differentiation of hfNSCs and enhances neurite outgrowth of immature neurons while promoting the expression of genes involved with forebrain development and neuronal differentiation, such as eomesodermin (EOMES, or T-box brain protein 2(TBR2)), doublecortin (DCX), and distal-less homeobox 2(DLX2) [206].

Neurodegenerative illnesses are fatal conditions that result in gradual deterioration of memory and motor skills [245]. There are several types of neurodegenerative disorders, including Pick’s disease, transmissible spongiform encephalopathies (TSEs), and Lou Gehrig’s disease (LGM). In various neurodegenerative diseases, age-related pathological changes in multiple brain regions have been associated with cognitive impairments [246]. In Alzheimer’s disease (AD) research, the hippocampus is the most affected area, with a reduction in its volume [247]. Parkinson’s disease (PD) patients with cognitive impairments have lower global and nodal phase linearity measurement (PLM) results in various temporal regions [248]. Amyotrophic lateral sclerosis (ALS) patients show a reduction in cortical volumes through voxel-based morphometry [249]. Due to the loss of specific neuronal populations, various neuropathological changes in neurodegenerative disorders are related to pain and decline in function [250]. Abnormal protein dynamics are also characteristic of neurodegenerative diseases, where disease-specific proteins can accumulate, mislocalize, or multimerize into fibrils and become toxic [251]. Several pathways, such as mTOR, MAPK, Ca^2+^, apolipoprotein E (APOE)-cholesterol, and high mobility group box 1 (HMGB1), are involved in the progression of these diseases, regulating the apoptosis, regeneration, and plasticity of neurons and microglia (Table 3). Although neurodegenerative diseases are typically sporadic, they can also be induced by mutations in proteins that are prone to aggregation. Mutations in the amyloid precursor protein (APP) gene cause familial Alzheimer’s disease [252]. In addition to genetic factors, ischemic conditions that cause damage to neuronal tissue can lead to secondary injury or progressive degeneration. Stroke is an example of an injury-triggered neurodegenerative disease [253]. Apart from abnormal protein and gene dynamics, these diseases share common features such as imbalanced antioxidant systems, mitochondrial dysfunction, neuroinflammation, impaired neurotrophin function, oxidative stress, and ineffective antioxidant defense [252,254,255].

Zingiberaceae plants and their active components have potential therapeutic applications in the treatment of neurodegenerative disorders. Previous studies have used a variety of extraction techniques to increase the yield of active compounds from ginger and curcumin, which can then be tested via in vitro and in vivo models of neurodegenerative diseases. These techniques include methods such as solvent extraction, steam distillation, and Soxhlet extraction, among others [256]. By using these extraction methods, researchers have been able to obtain higher concentrations of active compounds such as 6-gingerol and curcuminoids. Testing of these compounds in relevant disease models provides insights into their potential therapeutic applications in the treatment of neurodegenerative disorders. In some cases, standardized commercial formulations of the extracts have also been used. One such extract, curcumin, was found to promote neuronal regeneration in a rat model of parkinsonism induced by 6-hydroxydopamine (6-OHDA) by modulating the bFGF/NGF/TrkA/Hsp70 pathway. It increased the levels of superoxide dismutase (SOD) and glutathione peroxidase (GSH-Px), as well as the expressions of bFGF, NGF, and TrkA, while reducing the concentration of malonaldehyde (MDA) [212]. Another extract, the methanol extract of 6-paradol (6P) and 6-paradol-β-glucoside (6PG) from *Aframomum meleguet* seed, showed significant neurite outgrowth activity in a scopolamine-induced dementia mouse model of Alzheimer’s disease by increasing Ca^2+^ influx into the cells without activating ERK and CREB in the NGF-TrkA pathway [233]. The efficacy of *A. oxyphylla* (AO) in the treatment of Alzheimer’s disease (AD) has been demonstrated through research; the study identified 26 bioactive phytochemicals in AO that target 168 key molecules involved in the pathogenesis of neurodegenerative dementia. Yakuchinone B, 5-HYD, oxyhylladiketone, oxyphyllacinol, butyl-β-D-fructopyranoside, dibutyl phthalate, chrysin, yakuchinone A, rhamnetin, and rhamnocitrin were found to be the key phytochemicals that regulate the pathogenesis of neurodegenerative dementia in a multitargeted manner. Protein–protein interaction (PPI) analysis showed that the core targets, including AKT1, glyceraldehyde 3-phosphate dehydrogenase (GAPDH), TNF, IL6, catenin beta–1 (CTNNB1), mitogen-activated protein kinase 3 (MAPK3), vascular endothelial growth factor A (VEGFA), caspase-3 (CASP3), heat shock protein HSP 90-alpha (HSP90AA1), STAT3, estrogen receptor–1 (ESR1), and MTOR were highly ranked in the PPI network formed by all 662 AO targets [257]. Pharmacology and molecular docking approaches were used to investigate the mechanism of AO’s anti-AD activity, revealing that AO, particularly in its terpenes, possesses neuroprotective effects that regulate the synthesis, release, and transmission of neurotransmitters, as well as the formation and plasticity of dendritic spines and synapses in the nervous system [258]. Studies have demonstrated the significant neuroprotective activity effects of chloroform (CF) extract from the fruits of *Alpinia oxyphylla*. The CF extract was found to enhance cognitive performance, increase activities of glutathione peroxidase (GSH-px), and decrease levels of malondialdehyde (MDA), acetylcholinesterase (AChE), and amyloid-β (Aβ) in mice injected with Aβ1−42. The long-term treatment of CF also reversed the activation of microglia, degeneration of neuronal acidophilia, and nuclear condensation in the cortex and hippocampus. These results showed that CF ameliorates learning and memory deficits by attenuating oxidative stress and regulating the activation of microglia and degeneration of neuronal acidophilia to reinforce cholinergic functions [259].

In a mouse model of Alzheimer’s disease displaying cerebral amyloidosis and neuroinflammation, zerumbone, a sesquiterpene, was found effectively to improve behavioral impairments, reduce β-amyloid deposition, and attenuate neuroinflammation. The anti-inflammatory activity of zerumbone was observed in microglial cells, and it induced a phenotypic switch in microglia from a pro-inflammatory to an anti-inflammatory phenotype by inhibiting the MAPK signaling pathway. These findings suggest that the neuroprotective effects of zerumbone may be attributed to its ability to support the survival of neurons [260].

**Table 3:** Common pathways associated with neurodegenerative diseases.

Disease	Manifestation	Pathogenesis	Canonical Pathway	Reference
Age-related macular degeneration (AMD)	↑ level of complement components (C3, C3d, Bb, and C5, C5a)	Dysregulated metabolites in glycerophospholipid metabolism	mTOR	[261,262]
Transmissible spongiform encephalopathies (TSEs)	Presence of PrPSc and/or pathognomonic histopathological characteristics	The C-terminal region of the protein can cause amyloid formation by increasing the propensity of huPrP to aggregate due to the mutation T183A variant	Insulin signaling pathway	[263,264]
Pick’s disease	Atrophy of the frontal and anterior temporal lobes caused by an intraneuronal accumulation of aberrant protein inclusion bodies	↓ N-acetyl aspartate and glutamate	mTOR or Class III-PI3K/Beclin-1 complex	[265,266]
AD	↑ of β secretase expression causes, ↑ in amyloidogenesis↑ LPO under oxidative stress is significantly linked to neurotoxicity in AD Triggered by mitochondrial malfunction and oxidative stress	Mitochondria ↑ 4-HNE generated by damaged mitochondria Oxidative stress ↑ Ca^2+^ and ROS levels ↑ p-tau aggregates ↓ activities of antioxidants	APOE-cholesterol pathway	[253,267,268]
PD	α-Synuclein aggregation and build-up in the nervous system	↑ Protein aggregation of-α synuclein was accelerated by oxidative stress which causes the brain cells to die	Autophagy-lysosomal pathway (ALP) Ca^2+^ signaling MAPK mTOR	[269]
Progressive supranuclear palsy	Intracerebral accumulation of microtubule	Hyperphosphorylation of Tau protein	Insulin and neurotrophic factor pathways, Fyn kinase pathways in myelinating oligodendrocytes, PP1 pathway	[270,271,272]
ALS	Motor neuron degeneration	↑ β–catenin protein	Ca^2+^ pathway HMGB1 pathway	[273,274]

Common pathways associated with neurodegenerative diseases. (↑) denotes increment; (↓) denotes reduction.

### 1.5. Other Brain Disorders and the Role of Zingiberaceae Family Plants

Cerebral ischemia (lack of oxygen and blood flow to the brain) and brain trauma can cause loss of cellular function and tissue death. These conditions can result from various initial insults, including metabolic stress, biochemical and molecular events, and ionic perturbations [275]. Brain ischemic insult can be caused by factors such as severe reductions in cerebral blood flow, occlusion of cerebral and extracerebral vessel tissues due to thrombosis or embolism, or prolonged systemic hypotension [276]. Mechanisms of brain ischemic insult include glutamate toxicity, calcium toxicity, free radicals, nitric oxide, inflammatory responses, and endoplasmic reticulum or mitochondrion dysfunction [277]. Since one of the pathological characteristics of cerebral ischemia is represented by the neuroinflammation following microglia activation, it is necessary to find useful therapeutic strategies to reduce the neuroinflammatory responses in activated microglia. *Kaempferia parviflora* Wall. ex Baker provides neuroprotection for HT-22 neuronal cells with glutamate-induced cytotoxicity by increasing BDNF expression and reducing p-ERK levels [100]. If ischemia persists long enough, irreversible neuronal loss can occur, leading to an ischemic stroke. Neurons are unable to maintain their function without enough energy (ATP), which causes them to release their contents uncontrollably. This pathological release leads to an excessive amount of glutamate that binds to ionotropic glutamate receptors [-amino-3-hydroxy 5-methyl-4-isoxazolepropionic acid (AMPA) and N-methyl-D-aspartate (NMDA) receptors], causing over-excitation and allowing calcium to enter the cells. The influx of calcium activates downstream signaling pathways, leading to cellular damage and death [278].

Traumatic brain injury (TBI) is a brain insult caused by an external mechanical force, resulting in temporary or permanent impairment of cognitive, physical, and psychological functions, often accompanied by loss of or change in consciousness [279]. There are two types of neuronal tissue damage associated with TBI: primary and secondary injury. Primary injury occurs during the initial insult and affects localized areas, causing damage to brain tissues and vessels, and may lead to skull fractures or hematomas. Secondary injury, which follows primary insult, causes further tissue and cellular damage and progresses slowly over months to years [280]. The events during secondary damage include axonal degeneration, mitochondrial dysfunction, excitotoxicity, oxidative stress, and apoptotic cell death of neurons and glia [280]. This can lead to neurodegeneration and cognitive impairment. Patients with a history of TBI are at an increased risk of developing proteinopathy, which is a pathological feature of neurodegenerative disorders. Moderate to severe TBI also increases the long-term risk of stroke [281], particularly in older adults [282]. There is a need to find treatment to improve brain plasticity which could allow greater recovery after cerebral ischemia and injury. Pre-treatment with 6-gingerol from ginger reduced middle cerebral artery occlusion (MCAO)-induced iNOS, IL-6, and IL-1β protein production in microglia of C57/BL6J mice in an ischemia brain injury model [204]. *Zingiber officinale* Roscoe extract had a positive effect on inflammation-impaired SH-SY5Y cell viability by reduction of pERK levels and HDAC1 protein levels, inhibiting NF-κB signaling activation and reducing release of IL-1β, TNF-α, and IL-6 [283]. Increase in neurogenesis in a traumatic brain injury model using Sprague–Dawley rat brains by using curcumin powder was reported by Sun et al. (2020) [229]. P-coumaric acid from whole *Alpinia oxyphylla* Miq. ethanol extract improved poststroke cognitive impairment of adult hippocampal neurogenesis, and improved spatial learning, memory, and cognitive functions in post-MCAO ischemic rats [213]. Furthermore, repeated oral administration of *Alpinia katsumadai* extract protected neurons from ischemic damage in the hippocampus in a gerbil model of transient cerebral ischemia [235].

In an amnesia model, treatment of C57BL/6 mice with 6-gingerol increased the protein expression of BDNF, which was mediated via the activation of protein kinase B/Akt- and cAMP-response element binding protein (CREB) signaling pathway [210]. While amnesia is not necessarily considered a mental health problem in and of itself, it can be a symptom of underlying mental health conditions or brain disorders. For example, amnesia can be a symptom of depression, anxiety, dissociative disorders, or post-traumatic stress disorder (PTSD). Moreover, depression has been associated with combined frontal lobe and corpus callosum abnormalities [284]. The compound curcumin found in Zingiberaceae plants may also have antidepressant-like effects by modulating the PGC1α/FNDC5/BDNF pathway. It reversed depression and pseudodementia by reducing corticosterone levels and increasing hippocampal BDNF, 5-hydroxytryptamine (5-HT), dopamine (DA), and acetylcholine (ACh) levels in male Wistar rats [285]. *Zingiber purpureum* significantly induced neurite sprouting in PC12 cells, increasing the neurite length and number of neurites in primary cultured rat cortical neurons [286]. *Alpinia oxyphylla* Miq (fruits, ethanol extraction) demonstrated antidepressant-like effects on chronic unpredictable mild stress protocol on male Kunming mice. Another Zingiberaceae plant product, ginger-degraded collagen hydrolysate (GDCH) from ginger rhizomes reduced immobility time in the forced swim test while increasing GDNF and ciliary neurotrophic factor (CNTF) mRNA expression in the hippocampus of male ddY mice. This might have induced a neurotrophic effect on neural stem cells, astrocytes, and neurons in the stress model, exerting antidepressant effects [287].

On the other hand, volume loss in specific temporal and occipital regions has been linked to higher annual rates of memory deterioration [288]. Reductions in the tract integrity of white matter associated with ageing influence capacity for mental tasks [289]. An abnormal cortex is linked to schizophrenia [290] whereas abnormalities in perisylvian magnetoencephalography have also been observed in people with autism spectrum disorders [291]. For autistic neurodevelopmental disorders, 6-shogaol of ginger provides neuroprotection by reducing 4-hydroxy-2-nonenal (4-HNE) and myeloperoxidase, the biomarkers for autism [292]. Essential oil of *Alpinia zerumbet* leaves (EOAZ) reversed schizophrenia-like symptoms in male Swiss mice by increasing the release of IL6 and BDNF [208].

A polyherbal preparation from the Zingiberaceae family boosted the levels of expression of BDNF in a murine model of scopolamine-induced dementia [179]. It was reported that 6-shogaol increased choline acetyltransferase, choline transporter, and BDNF expression and decreased ROS production thereby decreasing ROS production through the BDNF/TrkB-mediated signaling pathway in H_2_O_2_-treated HT22 hippocampal neuronal cells [293] and exhibited anti-inflammatory activity by inhibiting NO, iNOS, PGE2, IL-1, TNF-, Cox-2, P38 MAPK, and NF-KB in BV2 and primary microglial cells that had been exposed to LPS [173,294]. These findings indicate the promising outcomes of 6-shogaol as a phytotherapeutic agent for the treatment of neurodegenerative illnesses. Zingiber purpureum Rosc., or bangle, exhibited neurotrophic effects in a murine model by increasing the number of Ki67-positive cells in the dentate gyrus of SAMP8 mice, showing neurotrophin-like activity by inducing neurite sprouting in PC12 cells [217]. Bangle extract increased the rate of neurite outgrowth from developing neurons and improved neuronal differentiation. Bangle extract also promoted the expression of genes involved in neurogenesis and the targets of WNT signaling. It also changed the histone modifications in human fetal neural stem cells (hfNSCs), making it easier for β-catenin to accumulate in their nuclei. To lessen the symptoms of neurological diseases and aid in neurorehabilitation, bangle may therefore be a compelling candidate [206].

Inhibition of Erk1/2 and Pkc had no effect on the (-) trans-banglene potentiation of NGF-induced neurogenesis [295]. Furthermore, the combined use of ginger and gabapentin demonstrated a neuroprotective effect as shown by overall improvement in the examined fetal brain tissues [296]. Gingerol-enriched ginger supplementation has also shown to be able to reduce neuropathic pain via the gut–brain axis [297]. Additionally, *Alpinia zerumbet* and *Alpinia oxyphylla* Miq. extracts alleviated schizophrenia-like symptoms and improved post-stroke cognitive impairment [208,213].

### 1.6. Zingiberaceae Family in Clinical and Toxicity Study

Although only a limited number of natural products have been tested in clinical trials, many compounds have shown promising properties in preclinical studies [298]. Ginger is listed as “Generally Recognized as Safe” (GRAS) by the United States Food and Drug Administration (FDA) and has been shown to possess significant potential for improving and preventing memory impairments. In preclinical and clinical studies [299], the use of ginger has shown potential as a cognitive enhancer in middle-aged women. A study involving 60 participants found that standardized ginger extract at a dose of 800 mg once daily for 2 months improved working memory and resulted in increased N100 and P300 amplitudes, decreased P300 latencies, and enhanced working memory, evaluated using computerized battery tests and the auditory oddball paradigm of event-related potentials [300]. However, for the prophylactic treatment of migraines, administering 200 mg of dry ginger extract (5% active ingredient) for three months did not provide significant benefits [301]. In a clinical trial involving children with generalized epilepsies, the addition of ginger to antiepileptic drugs (AEDs) such as sodium valproate or carbamazepine resulted in 87% of participants becoming seizure-free and all experiencing reductions in seizure duration and frequency [302].

Targeting the brain with drug delivery systems (DDS) is a promising strategy for improving the bioavailability and transport of compounds across the blood–brain barrier (BBB). However, despite the potential benefits, only a small number of natural compounds have been encapsulated in DDS for brain targeting. These include curcumin, which has been studied for its potential therapeutic effects on neurodegenerative disorders [298]. Several strategies have been proposed to overcome the challenges of curcumin’s low bioavailability, including the use of solid self-emulsifying drug delivery systems, solid dispersions, cyclodextrin inclusion complexes, prodrug synthesis, manipulation of the solid-state crystal structure, and micronization to increase the surface area of the drug for improved dissolution. Other approaches include the use of P-glycoprotein inhibitors such as piperine or quercetin, as well as nanoformulations. These strategies aim to enhance curcumin’s absorption and transport across the blood–brain barrier to improve its efficacy as a therapeutic agent for various diseases [303]. According to a meta-analysis study, curcumin was found to improve working memory performance more effectively than the placebo, although some gastrointestinal adverse events were reported. In the trials, six different curcumin formulations were tested to improve its bioavailability, including the use of turmeric essential oil, submicron curcumin particles, and solid lipid curcumin particles. However, there was no systematic comparison of these different formulations in terms of their clinical effectiveness on cognitive function [211]. Curcumin interventions have shown beneficial associations with gastrointestinal, neurological, and oral diseases. It was shown that 180 mg/day of Theracurmin, a highly absorbable curcumin formulation, improved memory and attention in non-demented people, as well as providing stabilization of cognitive functions in patients with Alzheimer’s disease (AD) and mild cognitive impairment (MCI) [304]. However, the interpretation of curcumin trial data is limited by considerable variation in the studies and small sample sizes (range: 34–96) [211,305]. It is also worth noting that the pharmacological and toxicological effects of curcumin are dose-dependent, and high doses may produce toxic and carcinogenic effects [306] as well as pro-oxidant effects [307]. Other reported side effects include mild nausea and diarrhea, and it can chelate iron and suppress hepcidin, leading to subclinical iron deficiency [308,309]. Additionally, a study using human hepatoma G2 cells found that curcumin had no mutagenic effect at a low concentration of 2.5 μg/mL, but it caused DNA damage in a dose-dependent manner at higher concentrations (10–40 μg/mL) [13]. However, human clinical trials using doses of 1125–8000 mg/day reported no toxic or adverse effects. These results indicate that synthetic curcumin is not mutagenic and is not toxic at doses up to 1000 mg/kg bw/day. Bacterial reverse mutation testing and mammalian micronucleus testing showed no evidence of synthetic curcumin causing mutations. Additionally, repeated oral dose studies lasting 14 to 90 days, with the highest dose tested being 1000 mg/kg bw/day, showed no toxicological concerns related to curcumin [310]. The toxicity of curcumin-loaded nanocomplexes (CNCs) was also found to be very low, at 0.27 and 0.54 g/kg bodyweight/day in mice and hamsters. In conclusion, the toxicity of high-dose CNC treatment was graded as very low, possibly due to the components of the nanocomplex [311]. Curcumin is generally considered safe and has demonstrated positive effects on multiple health outcomes in humans, with the advantages outweighing the disadvantages [312]. Combining curcumin with other dietary supplements, including piperine, α-lipoic acid, N-acetylcysteine, B vitamins, vitamin C, and folate, has been suggested to have a synergistic effect on cognitive function [313].

*Alpinia galanga* has demonstrated the ability to enhance cognitive performance in animals, although its psychostimulant properties and potential benefits for cognitive function in humans have not been extensively investigated. However, recent research suggests that *Alpinia galanga* (E-AG-01) may have a positive effect on mental alertness, and when combined with caffeine, it may improve sustained attention up to three hours after consumption [314]. These findings could potentially be relevant for elderly individuals, who commonly experience deficits in attention and cognitive function.

Methoxyflavones found in *Kaempferia parviflora* (KP) and *Curcuma longa* are known for their anti-inflammatory properties. However, due to their low bioavailability and first-pass metabolism, their efficacy in humans has not been fully established. Although some clinical trials have reported positive effects of *Kaempferia parviflora*, the evidence remains inconclusive due to the small size of the studies. Moreover, there were no reported harmful effects when a dose of 1.35 g/d of KP was administered.

Despite preclinical studies suggesting potential cognitive benefits of *Zingiberaceae* plants, limited clinical studies have been inconclusive due to various factors such as differences in plant origin, extraction methods, active compound strength, and low bioavailability of the herbal preparation. Future research should focus on improving the bioavailability of herbal preparations and their ability to cross the blood–brain barrier.

## 2. Conclusions

As evidenced by the literature review, Zingiberaceae family plants have an impact on neurotrophins and downstream signaling targets for neurocognitive function. The phenolic compounds from the Zingiberaceae family provide neuroprotection by preventing the NF-KB pathway from being phosphorylated (p-P65 and p-IκBα) and thus inhibiting NF-κB signaling activation and further reducing the release of proinflammatory cytokines. They might also prevent neuronal loss. Certain Zingiberaceae family members have demonstrated the potential to regulate neurotrophin levels by functioning as a modulator targeting the tropomyosin-related kinase (Trk) receptor or p75 neurotrophin receptor (p75NTR) to stop neurotrophin loss. They also enable neurons to renew and create a suitable environment for maintaining mature neurons. The Zingiberaceae family stimulates neurotrophin expression by modulating various pathways, including the mTOR, phospholipase C, Gamma 1 (PLC1), and p75NTR-mediated signaling pathways. The protective effect may occur through several pathways, mainly the P13KAKT-mTOR/STAT3 pathway, NF-κB, MAPK pathway, and cAMP-PKA-CREB signaling pathway. Neuronal regeneration promotion occurs via modulating the bFGF/NGF/TrkA/Hsp70, NGF-TrkA pathway and BDNF/TrkB/AKT signaling pathway, and the antidepressant-like effects of curcumin involve the PGC1α/FNDC5/BDNF pathway. Therefore, it can stop or slow the progression of dangerous and complicated neurodegenerative disorders. Additionally, based on their chemical makeup, plant phenolic compounds do not seem to be harmful. The BBB was found to be permeable to gingerol and shogaol, two of the bioactive compounds found in ginger, through passive diffusion [315].

## 3. Limitations and Future Direction

These studies should be interpreted considering their limitations. First, this is a narrative review and not a systematic review, hence it poses potential bias. Zingiberaceae plants are typically safe and exhibit potent neuroprotective effects in various neurodegenerative diseases and brain insults, even though they still have very few clinical therapeutic applications. Numerous studies indicate that dietary plant polyphenols from Zingiberaceae family plants are safe. While natural phytochemicals might be less harmful than newly developed synthetic drugs, their usage in traditional herbal medicines raises concerns regarding their reproducibility, specific medicinal effects, mechanism of action, and the identification of active ingredients, because such formulations are often prepared from crude materials, which makes it difficult to assess their effectiveness and identify the key components responsible for their medicinal properties. Despite various instances in the field of ethnopharmacology, preclinical in vivo investigations on phenolic substances that control neurodegenerative illnesses are still underrepresented. Knowledge of the mechanism remains in its infancy and the literature is scarce. There is a need to perform in-depth research to shed more light on the molecular interactions and crosstalk between the pathways involved, given the limited evidence on neurotrophic factor potentiation effects and the numerous pathways affected by *Zingiberaceae* family phenolic compounds. Low bioavailability is the biggest obstacle for phenolic compounds and largely limits their evidence-based adoption into clinical practice. Encouragingly, results from human studies, although limited in number, appear to support this preclinical basis, with improvements in cognitive performance and disease risk observed across healthy and disease states. By further understanding the pharmacokinetics and thereby improving delivery and bioavailability, researchers can exploit these phytochemicals’ abilities to halt age-related decline in cognitive function, brain diseases, and neurodegenerative disorders. Phenolic compounds that augment neurotrophins may not be a perfect treatment, but they may help to halt the progression of neurodegenerative illnesses or at least delay their development. A range of phenolic compounds from the *Zingiberaceae* family has been identified in this review and, as a result, they may offer interesting prospects for the treatment and symptom relief of brain illnesses and neurodegenerative diseases. For the observed relationships to be supported, nevertheless, more high-quality research is required.

## Figures and Tables

**Table 1:** Zingiberaceae phenolic compounds and their associated pharmacological activities.

Genera	Species	Vernacular/ Native Name	Bioactive Compounds	Pharmacology Action	Methods	Reference
*Zingiber*	*Zingiber cassumunar* Roxb. or *Zingiber purpureum* Roscoe	Phlai Bangle	Phenylbutenoids, cyclohexene derivatives, curcuminoids, sesquiterpenoids, benzaldehydes, naphthoquinones, monoterpenoids	Anticancer Antimicrobial	In vitro In vivo	[39,40,41]
	*Zingiber zerumbet*	Bitter ginger Lempoyang	Gingerol, shogaol, zerumbone, limonene, and humulene	Immunosuppression	In vivo	[42]
	*Zingiber montanum*	Bongelai Kunyit Bonglai	Curcuminoid	Antimicrobial Antioxidant Insecticidal (larvicidal and adulticidal) Anti-hypercholesterolemic	In vitro In vitro In vivo In vivo	[43,44] [45] [46] [47]
	*Zingiber officinale* var. Rubrum	Red ginger Halia bara	Vanilloids	Antioxidant Analgesic Antiemetic	In vivo In vivo Clinical trial	[48] [49]
	*Zingiber officinale* var Roscoe	Ginger Halia Halia Putih Haliak Layo Jahe putih besar Jahe badak Jahe gajah	6-Gingerol, paradols, 6-dehydrogingerdione	Antioxidant Antiinflammation Anti-obesity Antiemetic Cardiorespiratory protection Antidiabetic Anticancer Antimicrobial	In vivo In vivo In vivo In vitro Clinical trial In vivo In vivo In vitro	[12] [50,51] [52] [53] [54] [55] [56] [56]
	*Zingiber officinale* var Amarum	Emprit	Gingerol, shogaol, Zingiberene, beta-sesquiphellandrene, alpha-curcumene and trans-beta farnesene 1,8-Cineole, 1,8-p-methandiene arcurcumene, Bornyl acetate, camphene, citral, geranyl acetate, methylheptenone, z-citral, α-pinen and β-bisabolene	Antifungal Antimicrobial	In vitro	[50,50,57]
*Curcuma*	*Curcuma longa*	Turmeric Kunyit	Curcuminoids Curcumin-diferuloylmethane Demethoxycurcumin Bisdemethoxycurcumin	Anticancer Antioxidant Antiviral Anti-inflammatory Antibacterial Antifungal Antidiabetic Anticoagulant	In silico In vitro In vitro Clinical trial In vivo In vitro In silico Clinical trial	[58] [59] [60] [61] [62] [62] [63] [64]
*Alpinia*	*Alpinia zerumbet* *Alpinia zerumbet* (Pers.) Burtt. et Smith	Shell ginger	Aglycones, glycoside, benzoic and cinnamic acid derivatives	Antioxidant Anti-inflammatory Cardiovascular protective Antidiabetic	In vitro In vivo In vitro In vitro	[65] [66] [67] [68]
	*Alpinia conchigera* Griff	Wild ginger	Phenylpropanoids	Antimicrobial Antitumor Anti-inflammatory	In vitro In vitro In vitro	[69] [70] [71]
	*Alpinia galanga* (L.) Willd.	Galangal Lengkuas Siamese ginger	Diarylheptanoids, phenylpropanoids, and glycosides	Antioxidant, Antineoplastic Anti-inflammatory & Antidiabetic	In vivo In vitro In vitro	[72] [73] [74]
	*Alpinia officinarum*	Lesser galangal	Galangin- 3-hydroxyflavone flavonoids, diarylheptanoids, essential oils, phenylpropanoids, glycosides	Anti-inflammatory Antioxidant Anticancer	In vivo In vitro In vitro	[75] [76] [77]
*Hedychium*	*Hedychium coronarium* *Hedychium spicatum*	Hardy ginger	Coronarin D, isocoronarin D, linalool, villosin	Antioxidant & antibacterial Antitumor	In vitro In vitro	[78] [79]
*Elettaria*	*Elettaria cardamomum*	Cardamom	α-Terpinyl acetate, α-terpineol limonene	Antimicrobial Anti-proliferative Antioxidant & antimicrobial Anti-inflammation Antidepressant	In vitro In vivo In vitro In vitro In vivo Clinical trial	[80] [81] [82] [82,83] [84] [85]
*Elettariopsis*	*Elettariopsis latiflora*	-	Flavonoids	Antioxidant	In vitro	[86]
*Etlingera*	*Etlingera elatior*	Torch ginger Kecombrang	Nonyl cyclopropane, 1-tetradecane, cyclotetradecane, cyclododecane, and 1-decene	Antioxidant Anti-inflammation & antidiabetic Nephroprotective	In vivo In vivo In vivo	[87] [88,89] [90]
*Boesenbergia*	*Boesenbergia rotunda* (L.) *Mansf. Kulturpfl.* (syn. *Boesenbergia pandurata* (Robx.) Schltr.)	Fingerroot ginger Temu kunci	Quercetin, p-coumaric acid, chlorogenic acid	Anti-bacterial Antioxidant & anticancer Antioxidant properties	In vitro In vitro In vitro	[91] [92] [93]
*Kaempferia*	*Kaempferia galanga*	Kencur	Terpenoids, phenolics, diarylheptanoids	Anti-inflammatory Antioxidant Anticancer Antibacterial Anti-angiogenesis	In vivo In vitro In vitro In vitro In vitro	[94] [95] [96,97] [98] [99]
	*Kaempferia parviflora* Wall. ex Baker	Black ginger	5,7-dimethoxyflavone, 3,5,7-trimethoxyflavone	Antiaging Antiadipogenesis Antioxidant and anti-inflammatory Antimicrobial Gastroprotective	In vivo In vivo In vitro In vitro In vitro	[100] [101] [102] [103] [104]
	*Curcuma aeruginosa* Roxb.	Temu Ireng	Flavonoids, terpenoids, steroids, phenanthrenes	Anticancer Antioxidant Antimicrobial Anti-dengue Immunostimulant Anthelmintic Anti-inflammatory Antiandrogenic Anti-nociceptive and antipyretic uterine relaxant	In vitro In vitro In vitro In vitro In vitro In vivo In vivo Clinical trial In vivo In vivo	[105] [106] [107] [108] [109] [110] [111] [112] [113] [114]

**Table 2:** Zingiberaceae modulating neurotrophic pathways.

Source of Zingiberaceae	Active Compounds	Bioactivities	Model	Outcomes	Reference
*Zingiber purpureum* (dried rhizomes, ethanol extraction)	Phenylbutenoid dimers	Cognitive enhancer	PC12 cells	↑ neurite sprouting in PC12 cells	[217]
*Zingiber purpureum*	Neocassumunarins 1 Neocassumunarins 2, phenylbutenoid dimer 3	Neuroprotection	PC12 cells	NGF-mediated PC12 cells were stimulated by compounds 1 and 2 to generate neurites.	[218]
*Zingiber mioga* Roscoe (fresh flower buds, water extract)	Whole extract	Memory- enhancing effects Synaptic plasticity	Male Institute of Cancer Research (ICR) mice Cultures of rat hippocampal astrocyte cells	↑ NGF levels ↑ pCREB/CREB	[219]
6-gingerol (Shanghai, China)	6-gingerol	Neuroprotection	Male Sprague–Dawley rats	Activation of PI3K/AKT pathway	[220]
6-Gingerol (powder, Chengdu, China)	6-gingerol	Neuroprotection	Male Sprague–Dawley rats	↓ cytokine and nitric oxide synthase 2 (NOS2) protein expression ↑ GFAP (hippocampus and cerebral cortex)	[221]
6-gingerol (powder, Germany)	6-gingerol	Neuroprotection	Wistar rats	↑ BDNF and NGF ↓ 8-hydroxy-deoxyguanosine (8-OHdG) ↓ Bax ↑ Bcl2	[222]
6-Shogaol (Osaka, Japan)	6-shogaol	Anti-inflammation	Male ICR mice	↑ production of NGF, postsynaptic density protein 95 (PSD95)	[223]
6-Shogaol	6-shogaol	Neuroprotection	HT22 cells	↑ choline acetyltransferase (ChAT) and choline transporter (ChTp)	[224]
6-Gingerol & 6- Shogaol (Osaka, Japan)	G-gingerol 6-shogaol	Neuroprotective Neuroinflammation	BV-2 cells Sprague–Dawley (SD) rats	↓ cytokines ↓ microglial activation	[225]
6-Shogaol (Beijing, China)	6-shogaol	Neuroprotection	Murine BV2 microglia cells	↓PGE2 ↓ nuclear factor of kappa light polypeptide gene enhancer in B-cells inhibitor, alpha (IκBα) phosphorylation and degradation	[226]
6-Shogaol and 6-paradol (Dr. Dong Yoon Shin, Gachon University)	6-shogaol 6-paradol	Neuroprotection	Female C57BL/6 mice	↓ tumor necrosis factor-α	[227]
*Curcuma zedoaria*	Curcumenol	Neuroprotection	Nucleus pulposus (NP) cells, Sprague–Dawley rats	volcano plot analysis: curcumenol ↓ TNF and interleukin 1 receptor-like 1 (IL1RL1) but ↑ C-X-C motif chemokine ligand 10 (CXCL10) heat map analysis: ↑ C-X-C motif chemokine ligand 1 (CXCL1) and nitric oxide synthase 2 (Nos2), and ↓ TRAF1, IL1RL1, TNF, and homeobox A6 (HOXA6) ↑ MMP family’s expression and prevented the NF-KB pathway from being phosphorylated (p-P65 and p-IκBα)	[228]
Curcumin powder (St.Louis, MO)	Curcumin	Neuroinflammation neurogenesis	Sprague–Dawley rats	↑ neurogenesis ↑ BDNF and TrkB phosphorylation ↓ levels of PI3K/Akt phosphorylation	[229]
*Curcuma xanthorrhiza* Roxb.	50 mg curcuminoids	Anti-inflammatory	Female, 20–59 years old	↓ TNF-α after treatment	[230]
*Curcuma longa* Curcumin nanoformulation	Curcumin Docosahexaenoic acid (DHA)	Neuronal survival and repair	Neuronal degenerating cell model SH-SY5Y cells	↑ TrkB expression	[231]
Curcumin powder (Zwijndrecht, The Netherlands)	Curcumin	Bactericidal Neuroinflammation	BALB/c mice	↓ TNFα, interferon gamma, (IFNγ) and IL12	[232]
*Aframomum meleguet* (seed, methanol extract)	6-paradol	Neuritogenesis	PC12 cells Scopolamine-induced dementia mice	↑ Ca^2+^ influx	[233]
*Alpinia oxyphylla* Miq. (whole, ethanol extraction)	p-coumaric acid (P-CA)	Neurogenesis and improve poststroke cognitive impairment	In vitro: oxygen glucose deprivation plus reoxygenation on neural stem cell (NSC)	↑ BDNF, TrkB	[213]
*Alpinia oxyphylla* Miq (fruits, ethanol extraction)	Chrysin Tectochrysin Kaempferide	Antidepressant	Male Kunming mice	↑ pCREB/CREB, pERK/ERK	[234]
*Alpinia katsumadai* (seeds, ethanol extraction)	Whole extract	Neuroprotectant	Male Mongolian gerbils	↑ BDNF immunoreactivity	[235]
*Kaempferia parviflora* Wall. ex Baker (Rhizomes, ethanol extraction)	Whole extract	Neuroprotection	HT-22 neuronal cells	↑ BDNF expression and ↓ of p-ERK ↓ nuclear apoptosis-inducing factor (AIF) fraction levels	[100]
*Kaempferia* *parviflora* (KP) (rhizomes, ethanol extract)	Methoxyflavones-rich residue (MRR) compound 1–9	Preventing and improving cognitive decline	PC12D cells	↑ cAMP response element (CRE)-mediated transcription	[236]

Different outcomes of Zingiberaceae modulating neurotrophic pathways. (↑) denotes increment; (↓) denotes reduction.

## Data Availability

All data are publicly available.
